# Draft Genome Sequence of *Pseudoalteromonas* sp. Strain NW 4327 (MTCC 11073, DSM 25418), a Pathogen of the Great Barrier Reef Sponge *Rhopaloeides odorabile*

**DOI:** 10.1128/genomeA.00001-14

**Published:** 2014-01-30

**Authors:** Jayanta D. Choudhury, Arnab Pramanik, Nicole S. Webster, Lyndon E. Llewellyn, Ratan Gachhui, Joydeep Mukherjee

**Affiliations:** a School of Environmental Studies, Jadavpur University, Kolkata, India; b Australian Institute of Marine Science, Townsville, Queensland, Australia; c Department of Life Science and Biotechnology, Jadavpur University, Kolkata, India

## Abstract

To date, only one marine sponge pathogen (*Pseudoalteromonas* sp. strain NW 4327) has fulfilled Koch’s postulates. We report the 4.48-Mbp draft genome sequence of this strain, which is pathogenic to the Great Barrier Reef sponge *Rhopaloeides odorabile*. The sequence provides valuable information on sponge-pathogen interactions, including the mode of transmission and associated virulence factors.

## GENOME ANNOUNCEMENT

Over 8,500 sponge species have been described globally (1), and these highly diverse constituents of marine benthic communities have immense ecological importance, including for their role in seawater filtration and benthic-pelagic coupling (2). Declining marine sponge populations caused by diseases affecting various sponge species have been reported in several geographic regions (3). However, there is currently limited knowledge on the causative agents, modes of transmission, and pathogen virulence mechanisms of sponge diseases. Despite several reports linking sponge diseases with bacterial pathogens (4–8), only one study to date has actually confirmed Koch’s postulates by corroborating strain NW 4327 as a primary pathogen of the Great Barrier Reef sponge *Rhopaloeides odorabile* (9). *Pseudoalteromonas* sp. strain NW 4327 was previously isolated from a heavily fouled and necrotic specimen of a Great Barrier Reef sponge, *R. odorabile*, collected at the Davies Reef (18°50.24′S, 147°37.59′E), Pacific Ocean, Australia, from a depth of 8 m (9). A collagenolytic enzyme that was supposed to increase the pathogenicity of strain NW 4327 against this sponge was later purified (10). The 16S rRNA gene sequence (GenBank accession no. FR839670) assigned this strain to the *Pseudoalteromonas* genus.

For the isolation of genomic DNA, strain NW 4327 (MTCC 11073, DSM 25418) was cultivated in BD Marine Broth 2216 for 24 h. Genomic DNA was purified with the GeneJET genomic DNA purification kit (catalog no. K0721, Thermo Scientific). The genome was sequenced on an Illumina MiSeq platform (Illumina, Inc., San Diego, CA). A total of 3,364,637 paired-end reads, with a mean read length of 251 bp, were obtained, which yielded a total of 874.81 million bases. The raw reads that contained Illumina adapters and Ns were removed. High-quality data (reads with an average Phred score of ≥20) were assembled using the MaSuRCA version 2.1.0 genome assembler (11). The assembly generated 129 contigs (N_50_ value, 173,396 bp), comprising 4,482,415 bases. The final assembly had approximately 195-fold coverage, with the largest contig being 489,475 bp in size. Annotation was done using the NCBI Prokaryotic Genome Annotation Pipeline version 2.0, resulting in 4,179 genes, among which 3,970 coding regions, 47 rRNAs, and 113 tRNAs were found. The G+C content was estimated to be around 40.9%. Additionally, annotation was done by submitting all the contigs to the Rapid Annotations using Subsystems Technology (RAST) server (12).

Further comparative genome analysis will provide insights into the ecotaxonomic characteristics vis-à-vis the virulence mechanisms through which this sponge pathogen is pathogenic to its host. To the best of our knowledge, this is the first reported draft genome sequence of a confirmed marine sponge pathogen.

### Nucleotide sequence accession numbers.

This whole-genome shotgun project has been deposited in DDBJ/EMBL/GenBank under the accession no. AZIO00000000. The version described in this paper is the first version, AZIO01000000.
